# Sensing Mechanisms of Rough Plasmonic Surfaces for Protein Binding of Surface Plasmon Resonance Detection

**DOI:** 10.3390/s23073377

**Published:** 2023-03-23

**Authors:** Treesukon Treebupachatsakul, Siratchakrit Shinnakerdchoke, Suejit Pechprasarn

**Affiliations:** 1 Department of Biomedical Engineering, School of Engineering, King Mongkut’s Institute of Technology Ladkrabang, Bangkok 10520, Thailand; 2 College of Biomedical Engineering, Rangsit University, Pathum Thani 12000, Thailand

**Keywords:** sensing mechanisms, surface plasmon resonance, quantitative-sensing performance, binding-kinetics sensitivity, surface roughness, sensitivity-enhancement mechanisms

## Abstract

Surface plasmon resonance (SPR) has been utilized in various optical applications, including biosensors. The SPR-based sensor is a gold standard for protein kinetic measurement due to its ultrasensitivity on the plasmonic metal surface. However, a slight change in the surface morphology, such as roughness or pattern, can significantly impact its performance. This study proposes a theoretical framework to explain sensing mechanisms and quantify sensing performance parameters of angular surface plasmon resonance detection for binding kinetic sensing at different levels of surface roughness. The theoretical investigation utilized two models, a protein layer coating on a rough plasmonic surface with and without sidewall coatings. The two models enable us to separate and quantify the enhancement factors due to the localized surface plasmon polaritons at sharp edges of the rough surfaces and the increased surface area for protein binding due to roughness. The Gaussian random surface technique was employed to create rough metal surfaces. Reflectance spectra and quantitative performance parameters were simulated and quantified using rigorous coupled-wave analysis and Monte Carlo simulation. These parameters include sensitivity, plasmonic dip position, intensity contrast, full width at half maximum, plasmonic angle, and figure of merit. Roughness can significantly impact the intensity measurement of binding kinetics, positively or negatively, depending on the roughness levels. Due to the increased scattering loss, a tradeoff between sensitivity and increased roughness leads to a widened plasmonic reflectance dip. Some roughness profiles can give a negative and enhanced sensitivity without broadening the SPR spectra. We also discuss how the improved sensitivity of rough surfaces is predominantly due to the localized surface wave, not the increased density of the binding domain.

## 1. Introduction

Surface plasmon resonance (SPR) is a guided electromagnetic phenomenon that occurs when electrons oscillate between a noble metal and dielectric when illuminated by an external electric field, for example, an incident light with a matching momentum [1,2]. The coupling between the incident photon and electron oscillation leads to optical energy absorption [3], resulting in a dark band in plasmonic reflectance spectra. The coupling of SPR is a strong candidate for real-time monitoring of binding interactions [4], leading to the development of sensors based on SPR along with numerous other optical sensing technologies. Examples of applications using SPR include host–pathogen detection [5,6], biochemical interactions [7,8], ultrasonic detection [9], refractive index sensing [10,11], voltage sensing [12], drug discovery [13,14], microscopic imaging [15], and therapeutic monitoring [16].

**Figure 1:**
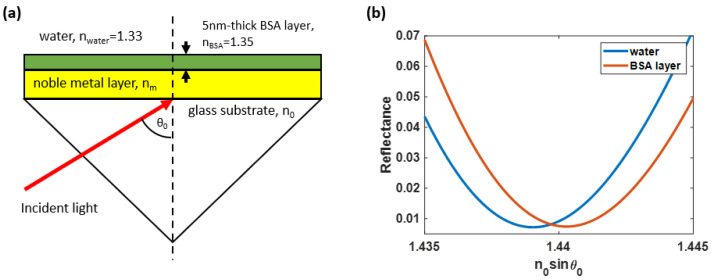
(**a**) The Kretschmann configuration consisting of a water-based sensing region, BSA binding layer, noble metallic film, a glass substrate, and incident light, and (**b**) the SPR sensor’s outputs consisting of reflectance curves, measuring from the water-based environment sensing region shown in the blue curve and a 5 nm-thick BSA coating layer in water shown in the red curve.

A widely adopted SPR-based sensor consists of a glass substrate with a refractive index of *n_0_*, a noble metal film with a refractive index of *n_m_*, and a sensing region with a refractive index of *n_s_*. This structure, also known as the Kretschmann configuration [17], is depicted in Figure 1a. There are several measurement schemes for SPR, including angular interrogation detection [18], wavelength scanning detection [19], intensity detection [20], and phase detection [21].

The angular interrogation detection [18] measures reflectance while varying the incident angle *θ_0_* at a fixed wavelength of *λ*, illuminating the thin plasmonic metal due to the explained light-matter interaction between photons and electrons. It manifests as a dark band in the reflectance spectra at a specific incident angle, known as the plasmonic angle *θ_sp_*, as depicted in Figure 1b. Furthermore, alterations on the metal film surface, such as molecular binding at an active site on the sensor surface, resulting in a subsequent alteration in the resonant coupling condition and a shift in the plasmonic angle, as depicted in Figure 1b.

Figure 1b shows the plasmonic angle shift from the water-backing environment with a refractive index of 1.33 to a 5 nm thick bovine serum albumin (BSA) with a BSA refractive index *n_BSA_* of 1.35 for an 80 mg/mL concentration [22] adhering to the metal surface, respectively. Figure 1b was calculated using the computational method explained later in Section 2.

Multiple studies [23,24,25,26,27,28] have reported that the surface profile, including the thickness and roughness of the metal film, significantly affects the performance of the SPR-based sensor for bulk refractive index sensing. For example, Kurihara et al. [27] explained that the SPR dips could be classified into large, small, and equivalent relationships between absorption and excitation frequency, depending on the frequency relationship between the optical property of the light source and the metal layer thickness. Furthermore, Hoffman et al. [26] demonstrated a significant change in dielectric functions and SPR wave vectors as the roughness amplitudes increased. Our recent publication [28] found that the sensitivity, full width at half maximum, intensity contrast, and plasmonic dip position for measuring the refractive index using an SPR-based sensor with rough gold films leads to a degradation of the sensor’s overall quality.

Several papers [29,30] reported that the sensitivity of binding kinetic measurement increases with the roughness level due to a larger surface area and higher surface protein in contact with the plasmonic metal. It is crucial to note that branching molecules, such as dextran, have been employed in binding surface preparation to increase the binding density. Unlike in rough plasmonic surfaces, for example, Liu et al. [30] employed a Ni seed layer on a silver-based SPR sensor to diminish the surface roughness, resulting in a smaller *FWHM*. In addition, Byun et al. [29] analyzed the localized surface plasmon resonance (LSPR) on a nanowire-based structure with different degrees of roughness. They also concluded that there was a change in the extinction peak amplitude caused by the LSPR effect.

Various surface treatment techniques have been employed as a postprocessing step for metal film fabrication to reduce surface roughness. Conventionally, the metal layer of the SPR-based sensor was manufactured using a sputter coating, which has a root-mean-square (RMS) roughness of 1.4 nm to 1.5 nm [31]. The metal film can be further smoothed through surface treatments, such as thermal annealing [32], helium ion beam [33], laser ablation [34], chemically grown single-crystalline gold [35], mica substrate utilizing [36], and chemical polishing [37,38]. Table 1 summarizes the roughness values for the mentioned treatment methods.

This paper theoretically investigated multiple roughness levels on a uniform gold sensor to analyze roughness’s effects on the SPR protein binding performance. This study has chosen the gold material due to its chemical stability and biocompatibility for biochemical and bioscience-based applications. A theoretical framework is proposed to analyze comparative sensing performance parameters, including protein binding sensitivity (*S*), the plasmonic dip position (*n_0_sinθ_sp_*), the full width at half maximum (*FWHM*), the intensity contrast (Δ*I*), the average plasmonic dip intensity (*I_sp_*), and the figure of merit (*FoM*). The theoretical investigation was based on rigorous coupled-wave analysis [39,40] and Monte Carlo simulation [41].

It is significant to note that the research differs from previous literature regarding protein binding to SPR sensors with different roughness levels. We systematically separate and quantify the two enhancement schemes: the LSPR and the increased binding density. The proposed method provides insight into the underlying performance enhancement of SPR sensors for binding kinetic measurement. In addition, we believe that the research provides a complete explanation of the impact of surface roughness on the molecular binding application of the SPR-based sensor performance and its limitations.

## 2. Materials and Methods

### 2.1. Rough Surface Model and Simulated Structures

The rough gold surface was constructed using numerical modeling with Gaussian random surfaces [42], which were adopted from Byun et al. [29], and characterized by varying maximum roughness height (*h*) and correlation length (*cl*) from 2 nm to 20 nm and 5 nm to 50 nm, respectively. The roughness factors were selected to cover SPR excitation and its cut-off positions. Different mode characteristics are present in the chosen roughness scope, including effects from LSPR and enhanced protein binding density, and these will be discussed later in the results section.

A function *s*(*x*) consisting of digital numbers 0 and 1 in a random sequence was first developed, where the term *x* indicated the spatial distance along the x-axis of the sensor to generate the rough surface. The function was then multiplied by the maximum roughness height, *h*. The generated profile was then converted to the frequency domain using Fourier transformation, expressed as *S*(*ω*) = *F*[*s*(*x*)], where *S*(*ω*) is the Fourier-transformed output, *ω* is the frequency domain axis, and *F* is the Fourier-transform operator. The *S*(*ω*) function was then multiplied with the Gaussian distribution function, *G*(*ω*) = *exp*(−*x*^2^/(*cl*^2^/2)). The *G*(*ω*) function essentially acts as a low-pass filter for the signal, where the term *cl* indicates the roughness correlation length. Next, the inverse Fourier transformation was applied to convert the signal back to the spatial domain signal and rearrange the function into a 200 columns rectangular contour. It is worth noting that higher *cl* values indicate a smoother surface. Therefore, a theoretical uniform surface is a Gaussian random surface with *h* equal to zero and *cl* approaching infinity.

The rough surface was then rearranged into a rectangular coordinate for rigorous coupled-wave analysis simulation by dividing the surface into multiple layers with a height of 0.1 nm each. The rough layer was then assembled with the uniform gold surface of the sensor. The rough surface structure profile consisted of three regions: (1) the yellow area beneath the surface indicating the plasmonic gold material with a refractive index of 0.18 + 3.43i [43], (2) the light green contour indicating the 5 nm-thick BSA binding layer [44] with a refractive index of 1.35, equivalent to 80 mg/mL concentration [22], and (3) the blue region indicating the water-based environment sensing area with a refractive index of 1.33. BSA was chosen as a substrate layer in this investigation due to its numerous applications, including cell culture and studies of biochemical reactions. Furthermore, BSA, including in biomedical research, has been widely adopted as a protein concentration standard in laboratory experiments.

**Figure 2:**
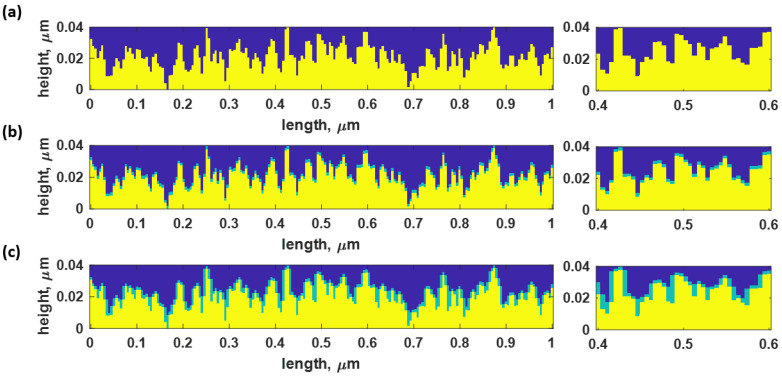
The Gaussian random rough surface for *h* 20 nm of and *cl* of 5 nm for (**a**) no BSA protein coating case (bare gold case), (**b**) BSA protein without sidewall coating, and (**c**) BSA protein with sidewall coating. The figures on the right show the zoomed-in version of rough surfaces for the three models.

The theoretical study was conducted using three sets of samples:

(1) no BSA protein layer case, as shown in Figure 2a;

(2) the second sample was a rough surface with only a BSA layer on top with no sidewall coating, as depicted in Figure 2b. This model indicated an equivalent amount of protein to the ideally smooth metal, allowing us to evaluate the enhancement due to the LSPR without the influence of the increased amount of protein due to the roughness;

(3) Conversely, the BSA layer in the third sample type was coated on top of the structure and its rough-surface sidewall, as illustrated in Figure 2c. The investigation of this structure aimed to replicate the protein solution binding to the entire surface, leading to an increased protein sample binding concentration. However, the amount of BSA in the third sample can be significantly denser than in the second sample when the surface is highly rough, as explained later in Section 3.3. Therefore, the increased protein and the LSPR can enhance the SPR response in the third sample, which the second model can suppress to quantify and separate the two enhancement mechanisms.

The sidewall coating behavior of proteins in nature was more complicated than in the simulation. Even though sidewall coating is typically used to prevent edges from coming into direct contact with water and to increase binding density, this assumption may not always be accurate. Even with sidewall coating, edges can remain in direct contact with water, which could affect the precision of the provided data. In such a way, the enhanced sensitivity and *FoM* will be between models (2) and (3). The results section will discuss that despite the absence of sidewall coating, roughness profiles can improve the SPR measurement due to the LSPR. However, many studies on polymer technology [45,46,47,48] assist in controlling protein fabrication. Saleem et al. [45] and Nuutinen et al. [46] investigate the application of polymeric material on resonant wave gratings (RWG). They utilized TiO_2_ as a cover layer on the gratings structure, and then the protein layer was uniformly bound to the material. Mayet et al. [47] published a similar study in which an oxide-nitride-oxide passivation layer evenly coated the sidewalls of the gratings. Zhang et al. [48] also conducted a theoretical study involving protein sidewall coating on a metal-layer-assisted double-grating (MADG) biosensor. These indicate that the experimental and theoretical information agree well and that the protein coating behavior can be controlled.

**Figure 3:**
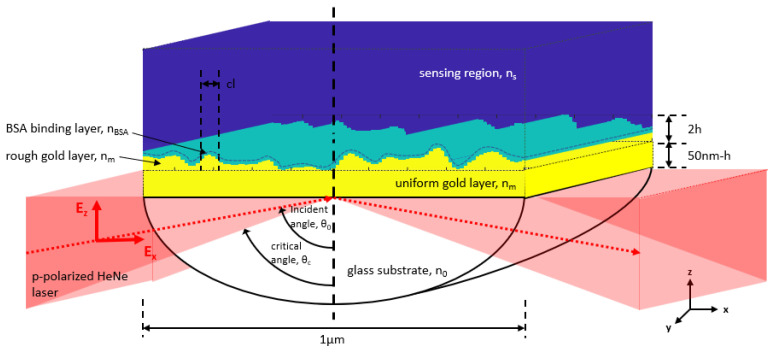
Simulated rough SPR sensor based on the Kretschmann configuration.

Figure 3 illustrates the overall simulation diagram, which consists of a BK7 glass substrate with a refractive index of 1.52, a uniform gold surface with a thickness of 50-*h* nm, a mean rough gold layer with a thickness and depth of *h*, a water-based environment sensing area with a refractive index of 1.33, a binding analyte (an 80 mg/mL concentrated BSA protein) with a refractive index of 1.35, and a thickness of 5 nm lining the top and sidewall of the rough surface layer, and a linearly p-polarized HeNe laser with a wavelength of 633 nm. The laser illuminates the gold film at an incident angle, scanning from the critical angle to 90°. According to the sampling theory, the unit cell length is 1 μm and consists of multiple rows with 1 nm each and 200 columns to ensure that the unit cell can provide different levels of the employed roughness with the minimum sampling feature size of 5 nm. The proposed 200 columns to represent the roughness in this study will be discussed and validated later in the results section.

As depicted in Figure 3, roughness profiles simulated in this study were simplified to a one-dimensional roughness profile, where the y-axis was treated as an infinite extension of the x-z plane. The Krestschmann configuration in this study was a prism-based Kretschmann configuration with TM polarization illumination. The TM incident electric field can be resolved in the x-z plane, with no electric field along the y-axis. That is the reason for the uniform y-axis in this study. It is essential to point out that this assumption may not hold if the SPR illumination is achieved using a high NA objective lens; there will be p-polarization components along the y-axis [49]. It will be shown in the results section that the proposed simplified simulation can give similar SPR reflectance spectra compared to the experimental results reported in the literature for the prism-based configuration and fabricated two-dimensional rough-gold sensors at different roughness levels.

### 2.2. Simulation of the Surface Plasmon Resonance Detection

This research employed the rigorous coupled-wave analysis (RCWA) function [40,50] and the Monte Carlo simulation to calculate the reflectance curve under a MATLAB 2022b environment, utilizing parallel computing and graphic processing toolboxes. In addition, 151 diffraction orders were applied to ensure that all results from the studied surfaces achieved convergence, which will be further discussed in Section 3.1.

**Figure 4:**
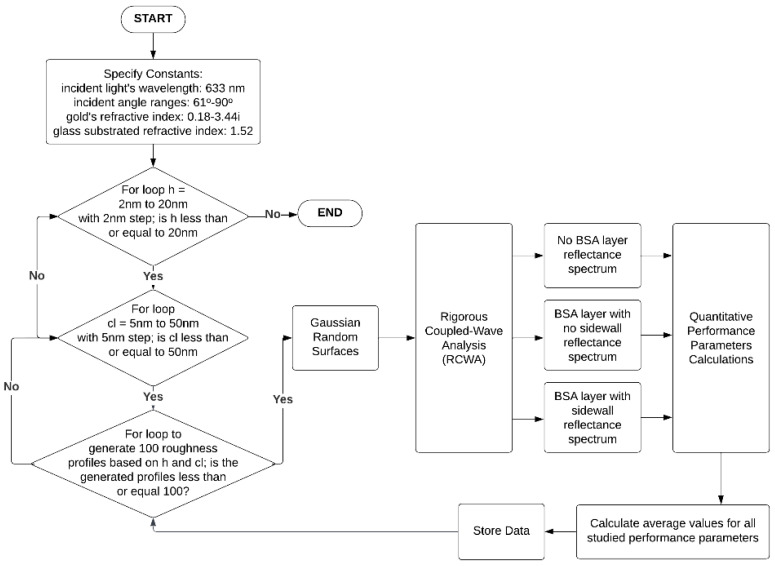
The flowchart of the overall simulation processes, including the rigorous coupled-wave analysis simulation, Monte Carlo simulation, and the performance parameters calculation.

Since a single pair of *h* and *cl* can generate multiple surfaces, the Monte Carlo simulation was applied to estimate an average response of 100 surface profiles for the three sample types. These responses from the rough surfaces generated by *h* and *cl* were then analyzed using the RCWA. Thus, each Monte Carlo simulation consists of performance factors’ values from 100 times RCWA simulation with a hundred different generated rough surfaces using a single value of *h* and *cl*, as illustrated in the flow chart in Figure 4. This procedure allows for a high accuracy and stability of the calculated performance parameters across all values of the roughness factors.

### 2.3. Quantitative Performance Parameters

The studied performance parameters of surface plasmon resonance detection for protein binding applications included sensitivity (*S*), full width at half maximum (*FWHM*), intensity contrast (Δ*I*), average plasmonic dip intensity (*I_sp_*), and figure of merit (*FoM*). This section provided definitions and descriptions of the presented performance factors; these parameters will be computed to explain the SPR sensing mechanisms for the protein binding in the results section.

**Figure 5:**
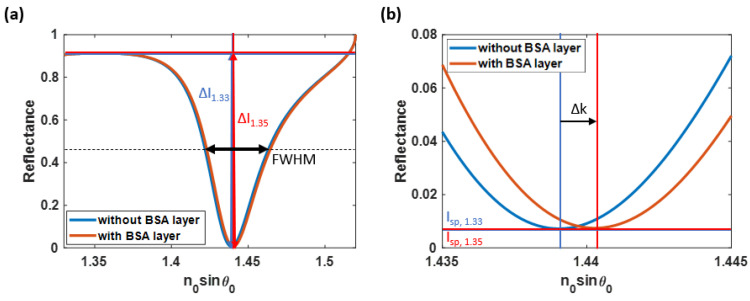
The calculation procedures of the discussed quantitative performance parameters for the ideal uniform gold surface sensor, including (**a**) Δ*I*, *FWHM*, (**b**) *S*, and *I_sp_*.

(1) The sensitivity (*S*) was calculated by dividing the change in the plasmonic wave vector (*k*), also known as the plasmonic shifting distance, by the product of the protein layer thickness and the difference in the binding region’s refractive indices, as shown in Equation (1) and Figure 5b.
  (1) $$S=\frac{\Delta k}{d\Delta n_{s}}=\frac{2\pi n_{0}\Delta sin\theta_{sp}}{\lambda d_{BSA}\Delta n_{s}}$$

The terms *n_0_*, *θ_sp_*, *d_BSA_*, *λ*, and Δ*n_s_* are the refractive index of the glass substrate (1.52), the plasmonic angle, the 5-nm BSA protein layer, the wavelength of the incident light (633 nm), and the difference in sample refractive indices of 0.02, respectively.

(2) The *n_0_sinθ_sp_* indicates the angular position at the minimum plasmonic intensity dip (*I_sp_*), which can be used to analyze the sensor’s detection range.
(3) The *FWHM* in this manuscript was calculated as the average width of the two plasmonic dips, measured at 50% of the reflectance spectra’s intensity, as expressed in Equation (2). Figure 5a illustrates the *FWHM* of the SPR curves, depicted as a black arrow considering the unsymmetrical nature of SPR dips.
  (2) $$FWHM=\frac{\Delta k_{nor,water}+\Delta k_{nor,BSA}}{2}$$

The terms $\Delta k_{nor,\;water}$ and $\Delta k_{nor,BSA}$ are the *FWHM* of the normalized reflectance curves, measured at half of the maximum magnitude when the refractive index of the sensing region was 1.33 and 1.35, respectively. In addition, several techniques can be applied to measure the *FWHM* of the unsymmetrical SPR dip, such as Lorentzian fit [51], Green’s function [52], and Horner’s curve fitting [53].

(4) The Δ*I* is one of the unique parameters which helps to determine the quality of the surface plasmon resonance-based sensor. It can be calculated as the average difference between the reflectance at the plasmonic angle and the intensity at the critical angle of the reflectance spectra, as expressed in Equation (3) and illustrated in Figure 5a.
  (3) $$\Delta I=\frac{\left|\Delta I_{water}\right|+\left|\Delta I_{BSA}\right|}{2}$$
  The terms Δ*I_water_* and Δ*I_BSA_* are the difference in the intensity at the plasmonic angles of the reflectance spectra detected when the 5 nm thick BSA binding layer is absent and present, respectively.
  (5) The *I_sp_* is another factor for considering the sensor’s performance. It can be defined as the average of the reflectance values at the plasmonic angles as expressed in Equation (4) and shown in Figure 5b.
    (4) $$I_{sp}=\frac{I_{sp,water}+I_{sp,BSA}}{2}$$

The terms *I_sp,water_*, and *I_sp,BSA_* are the intensity at the plasmonic angles of the reflectance spectra computed without and with the binding substrate.

(6) *FoM* encapsulates an overall sensor quality, usually defined as a ratio between the *S* and the *FWHM* [54], as expressed in Equation (5).
  (5) $$FoM_{1}=\frac{S}{FWHM}$$
  It is interesting to point out that the *FoM* depends on the detection mechanism for intensity-based measurement; the *FoM* is calculated based on the *S* and *FWHM* and can also be related to intensity parameters, such as Δ*I* and *I_sp_*, and considering the shot noise model [55], as expressed in Equation (6).
  (6) $$FOM_{2}=\frac{S\times \sqrt{\Delta I}}{FWHM\times \sqrt[{4}]{I_{sp}}}$$

### 2.4. Enhancement Ratio

Later in the result section, we will discuss that the *FWHM*, Δ*I*, and *I_sp_* are comparable for both the nonsidewall and sidewall models, indicating that the additional protein from the roughness does not affect these parameters. The *S* and *FoM* are the only performance parameters that can be affected by the sidewall protein. Here, the enhancement ratio denoted by *ER* compares the roughness models’ relative change in *S* and *FoM* to the ideal uniform gold sensor response. Furthermore, the LSPR wave, protein increment, and roughness effect will be quantified and classified using *ER* as expressed in Equations (7) to (9). Equations (7) to (9) show the calculations for the roughness enhancement effect, LSPR wave effect, and protein increase, respectively.

(7) $$ER_{S,roughness}=\frac{S_{sidewall}}{S_{uniform}}$$

(8) $$ER_{S,LSPR}=\frac{S_{non-sidewall}}{S_{uniform}}$$

(9) $$ER_{S,protein}=\frac{S_{sidewall}-S_{non-sidewall}}{S_{uniform}}=ER_{S,roughness}-ER_{S,LSPR}$$

The terms *ER_S,roughness_*, *ER_S,LSPR_*, and *ER_S,protein_* indicate the sensitivity enhancement ratios caused by roughness (the LSPR and the increased binding density), the LSPR wave effect, and increased protein binding density, respectively. Furthermore, *S_uniform_*, *S_sidewall_*, and *S_non-sidewall_* are the sensitivities obtained by the ideal uniform gold model, BSA with the sidewall model, and BSA without the sidewall model, respectively. The enhancement for the *FoM* can be calculated using the relative change in the *FoM*, similar to the sensitivity.

## 3. Results

### 3.1. Convergence Test for Extreme Cases

**Figure 6:**
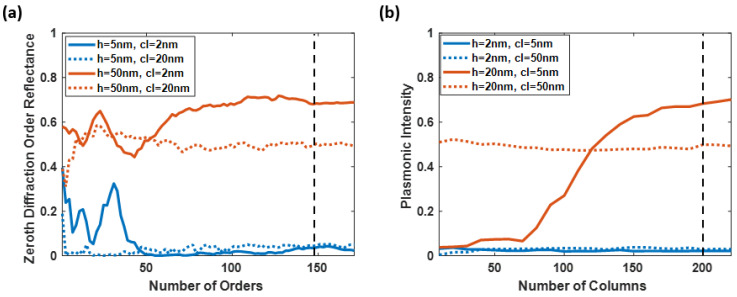
(**a**) The convergence test for the four extreme cases of the rough surfaces constructed with *h* of 2 nm and *cl* of 5 nm (solid blue line), *h* of 2 nm and *cl* of 50 nm (dashed blue line), *h* of 20 nm and *cl* of 5 nm (solid red line), and *h* of 20 nm and *cl* of 50 nm (dashed red line), tested with the number of diffractions orders up to 171, and (**b**) The convergence test on the simulated columns for the rough surfaces at the same four extreme cases, illustrated as average plasmonic intensities of the no BSA model, the nonsidewall BSA model, and the sidewall BSA model.

The convergence test on the optimal diffraction orders was employed in four extreme cases of the study, consisting of the rough surfaces generated using (a) *h* of 2 nm and *cl* of 5 nm, (b) *h* of 20 nm and *cl* of 5 nm, (c) *h* of 2 nm and *cl* of 50 nm, and (d) *h* of 20 nm and *cl* of 50 nm, as shown in Figure 6a. The test was performed using the rigorous coupled-wave analysis to calculate the p-polarized HeNe laser’s optical reflectance with multiple diffraction orders ranging from 1 to 171. The numerical fluctuation between the 149 and 151 diffraction orders was 7 × 10^4^, 0.002, 0.020, and 0.003 for the four tested surfaces. Employing a higher number of diffraction orders will surely increase the accuracy of the results; however, it also requires longer computing time and more resources. Therefore, the diffraction orders of 151 were employed in this study’s simulation cases with a numerical stability of 0.01 for the reflectance calculations.

The convergence test also inspected the adequate number of columns representing the rough surfaces, as shown in Figure 6b. The number of columns was investigated from 10 to 220 for the average plasmonic intensity of the no BSA model, BSA without the sidewall model, and BSA with the sidewall model. The tested roughness model was constructed based on the same four extreme cases. The plasmonic intensity’s differences between the 200 and 220 columns for (a) *h* of 2 nm and *cl* of 5 nm, (b) *h* of 20 nm and *cl* of 5 nm, (c) *h* of 2 nm and *cl* of 50 nm, and (d) *h* of 20 nm and *cl* of 50 nm was 7 × 10^4^, 0.002, 0.018, and 0.005 for the proposed three models. The following paragraphs will discuss how this roughness level can enhance protein binding. The analysis demonstrates that 200 columns are enough to simulate the roughness frameworks.

### 3.2. Comparison between the Proposed RCWA Simulation and Monte Carlo-Based Method and Reported Experimental Results in the Literature

One crucial question that we will discuss in this section is how accurate or how inaccurate the proposed RCWA simulation and Monte Carlo-based method can be compared to the experimental results. Yang et al. [56] reported experimental SPR reflectance measurements for different roughness levels for 632.8 nm incident wavelength and 57.5 nm gold films. These gold films were annealed using thermal annealing at four different temperatures at 100 °C to 400 °C for 15 min, allowing them to vary the roughness of the films. Their roughness parameters were measured using an atomic force microscope (AFM).

**Figure 7:**
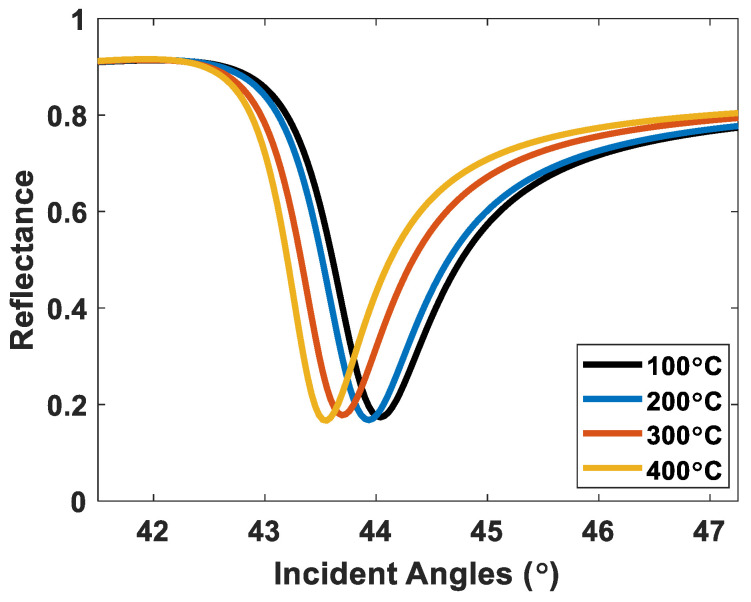
SPR reflectance spectra simulated using the proposed procedure at different roughness parameters reported by Yang et al. [56].

Figure 7 shows SPR reflectance spectra calculated using the proposed procedure using the roughness levels and the roughness heights reported by Yang et al. [56]. Table 2 describes the roughness levels, including the root-mean-square (RMS) roughness and the roughness heights, experimental plasmonic angles, and the plasmonic angles calculated from Figure 7. The plasmonic angles of the experimental results and the simulated SPR dips agree well, with slight discrepancies within 0.09 degrees. The slight error shown in Table 2 could be due to the gold used in the simulation may differ from the actual gold properties in the experiment.

### 3.3. Quantitative Performance Parameters of SPR Sensors at Different Roughness Levels

*Ideally smooth gold SPR-based sensor*

All quantitative performance parameters were initially calculated based on Equations (1) to (5) after the RCWA on the ideally smooth gold layer for the SPR-based sensor was simulated. As a result, the computed *S*, plasmonic dip position (*n_0_sinθ_sp_*), *FWHM*, Δ*I*, *I_sp_*, *FoM_1_*, and *FoM_2_* were 122.59 rad/µm^2^, 1.440, 0.41 rad∙RIU/µm, 0.90, 0.007, 299.00, and 980.66, respectively. These are the theoretical limits of the quantitative performance parameters of an ideally uniform SPR gold sensor calculated from Figure 5a.

*Rough gold surfaces*

**Figure 8:**
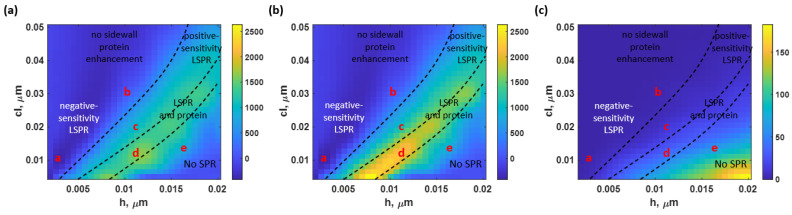
*S* of the (**a**) nonsidewall BSA model, (**b**) sidewall BSA model at different roughness levels in rad/µm^2^, and (**c**) the increase of the protein density due to sidewalls in percent as the surface roughness increased.

Figure 8a,b illustrate the *S* of the SPR on a nonsidewall BSA model and a sidewall BSA model to different roughness using RCWA and Monte Carlo simulation computed using Equation (1). Furthermore, the increment of the BSA binding domain, in percent, in the sidewall model compared to the BSA-on-top model at different roughness was shown in Figure 8c. Two gratings’ models with equivalent *h* and *cl* were generated using the same principle to calculate the protein increment. The additional sidewall protein can be calculated by subtracting the nonsidewall model from the sidewall model. Later, the percentage of protein increment can be determined by dividing the sidewall protein by the nonsidewall model’s protein amount. According to the contour representation, the protein difference remained negligible at the low-to-moderately rough surface. However, the increased amount of protein due to the roughness significantly increased as the roughness height, *h*, escalated to over 10 nm for a short correlation length, *cl*.

**Figure 9:**
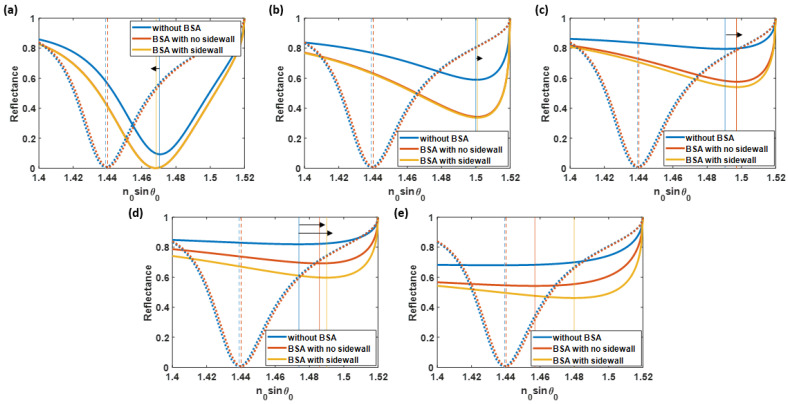
(**a**) the reflectance spectra with a negative sensitivity at the operating point ‘a’, (**b**) degraded-sensitivity reflectance curves at the operating point ‘b’, (**c**) enhanced-sensitivity reflectance spectra at the operating point ‘c’, (**d**) highly enhanced-sensitivity reflectance spectra at the operating point ‘d’, and (**e**) the reflectance curves at the highly rough surface where the sensitivity cannot be computed at the operating point ‘e’. Note that the dotted curves indicate the plasmonic angles of the ideal uniform gold cases and the black arrows indicate the plasmonic angle shift direction.

The *S* can be classified into four major regions:

(1) The “no sidewall protein enhancement” corresponds to the region where the letters ‘a’ and ‘b’ are in Figure 8. This area indicated the roughness levels in which the sidewall BSA was negligible. The protein lying on top of the rough surfaces overlapped the sidewall protein. Moreover, two types of SPR results were investigated in this region: the negative-sensitivity LSPR and the degraded-sensitivity reflectance spectra, as illustrated as the operating point ‘a’ and ‘b’, respectively. The negative plasmonic dip shifting means the plasmonic angle shifts towards a lower plasmonic resonance angle when the sample refractive index increases. It is established that this is due to the adverse diffraction orders of gratings or scattering surfaces [57]. The reflectance spectra at the operating point ‘a’ (*h* and *cl* of 3 nm and 10 nm, respectively), as shown in Figure 9a, had an *S* of −379.88 rad/μm^2^; approximately threefold of the magnitude of the *S* acquired from the ideal uniform sensor. Within the same region but at rougher surfaces, with *h* of 10 nm and *cl* of 30 nm (operating point ‘b’), the *S* returned to the positive value but degraded to 85.31 rad/μm^2^, indicating a 30.41% decrease in the sensitivity. The averaged reflectance spectra calculated from 100 structure profiles at these roughness levels are shown in Figure 9b;
(2) The “positive-sensitivity LSPR” only described the *S* affected by the LSPR. Here, the additional protein was presented but had an insignificant effect on the *S*. The reflectance curves spectra at the operating point ‘c’ obtained at *h* of 12 nm and *cl* of 20 nm indicated the enhanced *S* due to the LSPR wave effect only. Figure 9c illustrates the reflectance spectra at this location. In addition, the two employed models’ curves in this roughness level did not look significantly different due to the small increment of the BSA amount;
(3) The “LSPR and protein” was investigated as the roughness rose. In this region, *S* of the SPR spectra can improve due to the localized LSPR effect and the presence of an additional protein. The models indicate a significant difference in sensitivity values at the operating point ‘d’, located at *h* of 9 nm and *cl* of 8 nm. For the non-sidewall BSA model, the *S* of 1191.13 rad/μm^2^ was investigated. In contrast, the BSA sidewall model achieved a significantly higher *S* of 1608.02 rad/μm^2^, having a 35.00% increase from the nonsidewall BSA model, as shown in Figure 9d. The binding sensitivity enhancement due to the LSPR was 9.72 times at this roughness level.
  In contrast, the BSA sidewall model enhanced the *S* by 13.12 times more than the ideally smooth gold sensor, indicating that the *S* enhancement due to the increased protein was 3.68 times. In addition, this crucially high *S* was due to the deterioration of a plasmonic dip structure, resulting in a lower *n_0_sinθ_sp_* for the nonprotein coated model, which will be explained in the next part. The *S* enhancement factor due to additional protein agreed with the estimated protein concentration at the roughness level, as shown in Figure 8c;
(4) At the extreme roughness levels (near the bottom right corner of the contour in Figure 8), the plasmonic dip structure deteriorated, as indicated by the “No SPR” region, due to the scattering loss [58] of the rough surface. The propagation length of surface plasmon polaritons is strongly distorted due to roughness [59], resulting in an undetectable region, as indicated in Figure 9e for the operating point ‘e’ (obtained at *h* of 16 nm and *cl* of 15 nm). The plasmonic dips have virtually no intensity contrast. The SPR reflectance dips weakly maintained their structure and distorted at the higher roughness. The increase in protein quantity due to roughness was not the only reason for the improved *S* but also the enhancement of the LSPR from the roughness peaks. There is a substantial tradeoff between the roughness level, the sensitivity, and the scattering loss of the SPR dip.

**Figure 10:**
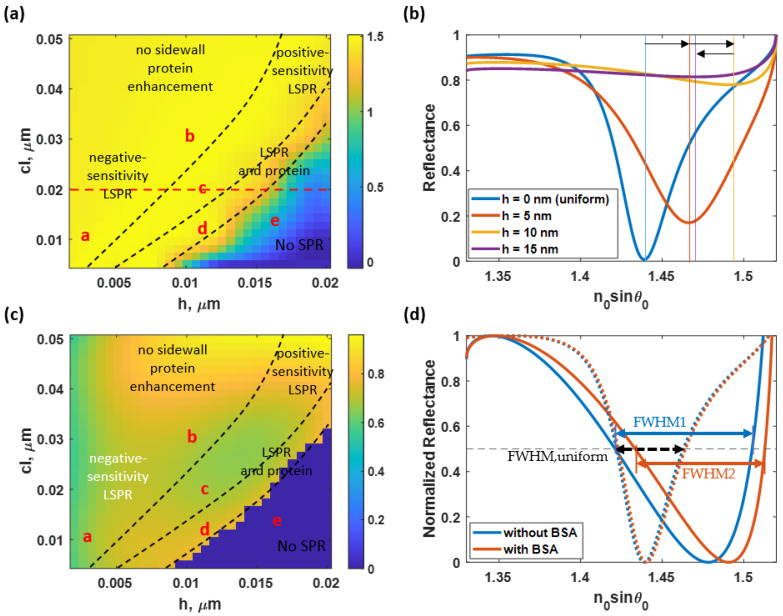
(**a**) the average plasmonic dip location, *n_0_sinθ_sp_*, for the three models at different roughness levels, and (**b**) the reflectance spectra from the nonprotein coated model at *h* of 0 nm (ideal smooth), 5 nm, 10 nm, and 15 nm, and the equivalent *cl* of 20 nm. (**c**) The *FWHM* of the SPR detection at different degrees of roughness and (**d**) reflectance spectra for the rough gold surface of *h* and *cl* of 18 nm and 40 nm, respectively, compared to the uniform gold surface. Note that the black arrows in (**b**) indicate the plasmonic angle shift direction when the *h* increased.

Figure 10a illustrates the average *n_0_sinθ_sp_* of the SPR dip positions calculated from the BSA without the sidewall model, the BSA with the sidewall model, and the without BSA coating model. The parameter can be used to identify the sensor’s detection range. An increased surface roughness could result in a higher plasmonic angle and a shorter detection range. Moreover, a higher *n_0_sinθ_sp_* results in a bigger numerical aperture (NA) requirement of the illumination lens for angular scanning applications. Figure 10b compares the reflectance curves from the model without the BSA coating and their plasmonic dip position at *h* of 0 nm (ideal smooth), 5 nm, 10 nm, and 15 nm, and the equivalent *cl* of 20 nm. As the theoretical uniform surface became rougher to 10 nm, the *n_0_sinθ_sp_* shifted gradually. At this *cl*, the shifting distances from *h* of 0 nm to 5 nm and *h* of 5 nm to 10 nm were very similar at approximately 0.05. However, when the *h* is increased to 15 nm, the *n_0_sinθ_sp_* moves slightly backward due to the deterioration of the plasmonic structure due to surface roughness.

Figure 10c shows the *FWHM* of the reflectance spectra at the simulated rough surfaces. The parameter was computed using Equation (3). The *FWHM* results for the sidewall and no sidewall cases were similar; therefore, only the *FWHM* obtained from the non-sidewall BSA model was presented to shorten the manuscript. The growth of roughness strongly broadens the size of the reflectance spectra. At slightly rough surfaces with an *h* lower than 5 nm, the *FWHM* was affected by *h* only. For instance, operating point ‘a’ had an *FWHM* of 0.58 rad∙RIU/µm, 41.46% higher than the ideal uniform sensor’s *FWHM*. However, when *h* was greater than 5 nm, *cl* significantly affected the alternation in *FWHM*. At rough surfaces, with a *cl* less than 35 nm, the reflectance spectra may have a deteriorated structure, reducing Δ*I*. The crucially low Δ*I* can result in a slightly lower *FWHM* than the region with a larger *cl* (smoother surface). These changes in Δ*I* will be discussed further in the following paragraphs. In addition, operating points ‘b’, ‘c’, and ‘d’ were in this region.

On the other hand, at the operating point ‘e’, there was a crucial loss in the SPR structure, resulting in an unidentified *FWHM*. However, the intensity depth *I_sp_* of the SPR dips considerably deteriorated due to the scattering loss compared to the smooth sensor surface. Apart from the mentioned regions, it can be increased up to 0.83 rad∙RIU/µm at an *h* and a *cl* of 18 nm and 30 nm, respectively, which was approximately two times wider than the *FWHM* of the uniform gold SPR detection, as shown in Figure 10d. The rougher surface leads to stronger scattering and a broader SPR dip.

**Figure 11:**
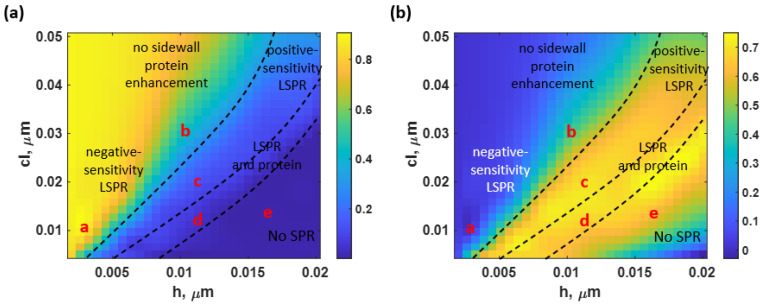
(**a**) the average Δ*I* and (**b**) the average *I_sp_* of both the nonsidewall BSA and the sidewall BSA models.

Figure 11a,b indicate the average Δ*I* and *I_sp_* at various roughness levels. The results obtained from the BSA without the sidewall model and the BSA with the sidewall model were slightly distinct, with a maximum difference of approximately 0.1 for both parameters, as shown in Figure 11a,b. Therefore, only the average values were reported to shorten the manuscript. In addition, this indicated that the presence of a sidewall protein does not significantly affect the change in Δ*I* and *I_sp_*. However, the surface roughness still substantially impacted the sensor’s output. At an operating point ‘d’, for instance, the average Δ*I* was 0.089, which was 10.08 times less intensity contrast than Δ*I* achieved by the theoretical uniform gold sensor. Furthermore, the average *I_sp_* crucially rose to 0.74 (105.71 times compared to the ideally smooth gold sensor’s *I_sp_*).

**Figure 12:**
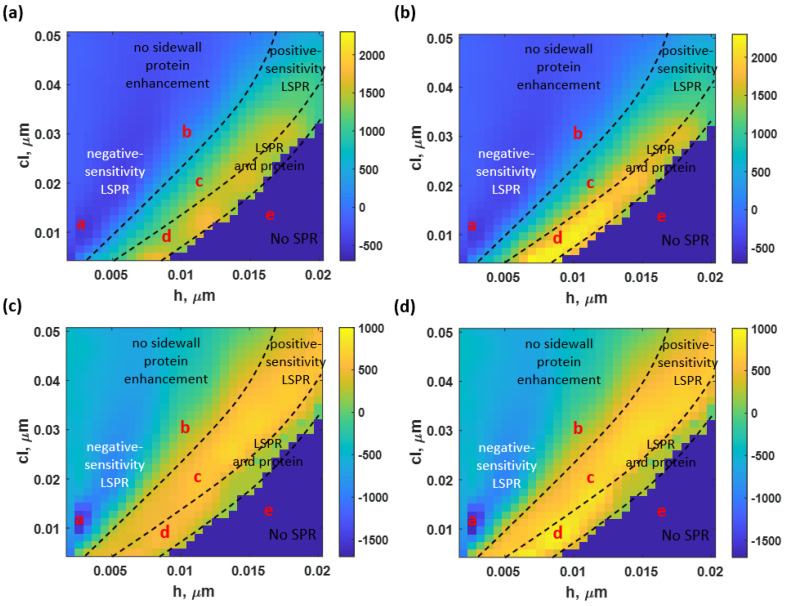
(**a**) the *FoM_1_* of the BSA without the sidewall, (**b**) BSA with the sidewall model; (**c**) *FoM_2_* for SPR detection [55] obtained from the BSA without the sidewall, and (**d**) BSA with the sidewall model.

The reported quantitative performance parameters were combined to compute the *FoM*, calculated based on Equation (5), at different roughness levels for BSA with no sidewall model and BSA with a sidewall model, as shown in Figure 12a,b, respectively. Figure 12c,d show *FoM_2_* calculated using the intensity-based *FoM* expressed in Equation (6) [55]. The protein enhancement due to the roughness neither distorts nor enhances the LSPR in the range of studied rough surfaces.

The FoM_1_ in Equation (5) depends mostly on how far the SPR dip moves *S*, not the optical intensity. Moreover, the results can be interpreted as the degradation and enhancement trends due to LSPR relying on surface roughness. In contrast, the additional protein can enhance the performance parameters, including *FoM*, at the expense of a more demanding NA requirement. The sensor achieved its maximum *FoM_1_* and *FoM_2_* of 1777.81 and 2400.02 at an *h* of 9 nm and a *cl* of 8 nm (operating point ‘d’) for the nonsidewall BSA and sidewall BSA models, respectively. The reflectance spectra at this point had a significantly increased *S* and a slightly dropped *FWHM* due to both the LSPR effect and the protein increment, resulting in the crucially high *FoM_1_*. In addition, a negative *FoMs* region resulted from the negative *S* values. The *FoMs* in some of these reported operating positions could surpass the sensing capability of the ideal uniform gold sensor. For instance, the operating point ‘a’ had an *FoM_1_* of −654.97, about two times higher than the theoretical smooth gold sensor’s *FoM_1_*. Other operating points’ *FoMs* and other performance parameters are indicated in Table 3.

The SPR detection’s *FoM_2_*, calculated involving *S*, *FWHM*, Δ*I*, and *I_sp_*, had similar results, as illustrated in Figure 12c,d for the BSA without the sidewall and BSA with the sidewall model, respectively. The highest *FoM_2_* for the SPR detection was at the same operating point as the *FoM_1_*, with an *FoM_2_* of 616.84 for the nonsidewall BSA model and 1020.33 for the sidewall BSA model, respectively. Therefore, the surface roughness can slightly improve the overall sensing performance in the intensity detection of the SPR-based sensor for the binding kinetic measurement.

Table 3 compares the seven investigated performance parameters obtained from the five operating points. It is seen that the surface roughness can result in an enhancement (operating points ‘c’ and ‘d’), a degradation (operating point ‘b’), or a complete deterioration (operating point ‘e’) of the SPR detection due to both the LSPR effect and the additional protein. The *S* can be significantly increased with highly rough surfaces in exchange for the substantial deterioration in the application relying on intensity detection. There is a tradeoff between the *S* enhancement due to the increased protein concentration, in other words, binding density, and the *FWHM* of the SPR reflectance dip. Despite this limitation, the negative-sensitivity detection (operating point ‘a’) could also be an efficient alternative for utilizing the SPR intensity-measurement applications due to its improved sensitivity and slightly degraded *FWHM*.

The *ER* and the increased protein density and surface roughness effects on the *S* and *FoMs* are summarized in Table 4. The *ER* for the roughness effect, the LSPR effect, and the protein increment were calculated based on Equations (6) to (8). The only factor affecting the sensor’s performance for operating points ‘a’ and ‘b’ was the LSPR since the protein density was negligible, as explained in the earlier section. Note that some *ERs* for the operating points ‘b’, ‘c’, and ‘d’ are less than 1.00, indicating that the roughness level experienced a degradation effect. On the other hand, the additional protein and the LSPR affected the operating points ‘c’ and ‘d’.

**Figure 13:**
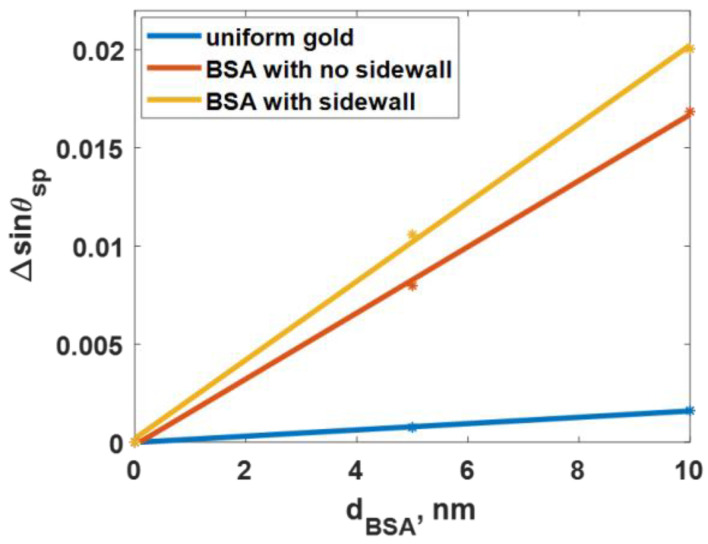
The Δ*sinθ_sp_* for different deposited thicknesses of BSA from 0 nm (bare gold) to 10 nm for ideal smooth gold surface, rough gold surface with *h* of 9 nm, and *cl* of 8 nm for the no sidewall and sidewall coating cases.

The roughness level of the *h* of 9 nm and the *cl* of 8 nm, corresponding to the highest *FoM_1_* and *FoM_2_* responses discussed earlier, was further analyzed to validate their sensing response. One crucial performance parameter for protein kinetics is to check whether the sensor has a linear responsivity for different protein concentrations, such as the thickness deposited on the sensor surface. Figure 13 compares the linear responsitivity of the three models for the *h* of 9 nm and the *cl* of 8 nm roughness sensor computed in this study for the different deposited thicknesses of BSA from 0 nm (bare gold) to 10 nm. The three models had linear responsivity covering the protein thickness, with R-square values for the linear equation fitting of 0.9985, 0.9990, and 0.9990 for the ideal smooth gold surface, the rough surface with no sidewall protein coating, and the rough surface with a sidewall protein coating, respectively. The slopes (Δ*sinθ_sp_/d_BSA_*) of the three linear lines were 8.13 × 10^−4^, 1.69 × 10^−3^, and 2.01 × 10^−3^, for the three models, which can be converted to the *S* in Equation (1) and it was found that the *S* values were 122.59 rad/μm^2^, 1202.43 rad/μm^2^, and 1595.30 rad/μm^2^ for the uniform gold surface, the BSA without the sidewall model, and the BSA with the sidewall model for the 5-nm thick BSA layer case. The *S* values agree with the *S* discussed in Table 3, indicating that the rough surface can provide a linear responsivity covering 0 nm to 10 nm. Having mentioned that the uniform sidewall protein coating assumption may not be accurate, Figure 13 provides a feasible approach to predicting the expected *S* if the protein does not fully cover the sidewall. The partial sidewall coating’s *S* will be between the no sidewall coating case (red line) and the uniform sidewall coating (yellow line).

## 4. Discussion

### 4.1. Effect of LSPR and Sidewall Protein from Roughness

Table 4 shows that the wave effect was responsible for most of the impact on sensor quality. For *S* and *FoM_1_*, all roughness levels that preserve the SPR spectrum have a percent impact of wave effect greater than 70%. The *FoM_2_*, which considers the Δ*I* and *I_sp_*, experienced a greater impact from the sidewall protein. Furthermore, the percentage of *S* and *FoM_1_* enhancement of the operating points ‘c’ and ‘d’ in Figure 8c were close to the percentage of protein increment due to roughness. The calculations using the explained framework using the RCWA and Monte Carlo simulation agree well with the additional protein calculated directly from the grating profile. The agreement provides an independent measurement of the protein density enhancement due to the roughness and tells us that the enhancement due to the LSPR and the increased protein are independent. This investigation implies that increasing the protein amount increases the *S* and the *FoMs* at the same rate.

In this paper, we have explained and provided insight into the detection mechanisms of rough surfaces. Rough surfaces can enhance the binding domain density, *S*, and *FoM_1_* with an expense of *FWHM* and optical intensity contrast of the SPR dip. It is essential to point out that the advantage of rough surfaces is that they provide only a single SPR dip or SPR mode from scatterings of surface plasmon polaritons (SPPs). In other words, an average effect of scattered SPPs and LSPR hotspots at the sharp edges [60]. However, rough plasmonic surfaces also have drawbacks, including fabrication repeatability, nonlocalized heat sources due to the localized hotspots at the sharp edges, and nonuniform distribution of the SPPs, making it difficult to be employed in optical imaging applications. Another alternative approach to achieve a similar enhancement of the binding domain density, *S*, and *FoMs* is plasmonic grating. Plasmonic gratings allow the SPPs to propagate in a well-defined path and support the additional sidewall protein. The plasmonic gratings can support and excite multiple modes due to the diffraction orders leading to a limited detection range due to crosstalks and overlapping modes [61].

### 4.2. Intensity and Phase Detection Schemes

**Figure 14:**
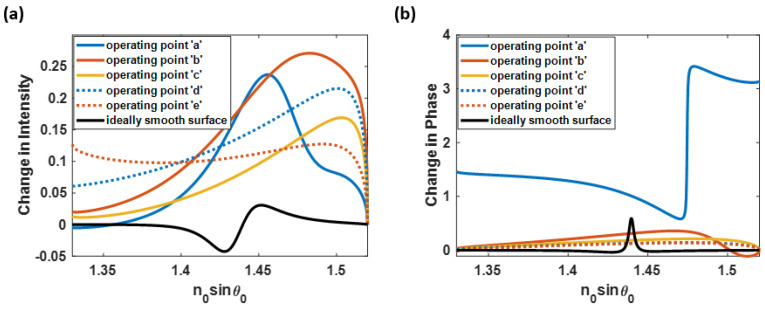
(**a**) Intensity detection and (**b**) phase detection of the operating point ‘a’, ‘b’, ‘c’, ‘d’, and the theoretically uniform surface SPR-based sensor.

Aside from angular interrogation, the SPR measurement can be done using the wavelength scanning method through intensity and phase detection, as in the angular interrogation investigated in this paper. In addition, these schemes are not affected by scanning wavelengths and angles. Here, the five discussed operation points and the ideal uniform surface sensor were analyzed for their intensity detection and phase detection performances, as illustrated in Figure 14. Figure 14a shows that the roughness profiles can generate a more significant optical intensity contrast than the theoretically smooth plasmonic layer case. For instance, the operating point ‘b’, which has significantly low sensitivity; on the other hand, can provide a crucial change in the plasmonic intensity of 0.27. The phase detection results have an alternative outcome, as shown in Figure 14b. Only the operating point ‘a’ (negative sensitivity SPR) has an outstanding performance in phase detection, while the other operating positions have degraded their phase responses. According to the analysis, the negative sensitivity measurement of SPR with a rough surface can be a strong candidate for binding kinetics measurement.

### 4.3. Field Plots on Exemplary Optical Structures

Light possesses both an electric field and a magnetic field, and this property of electromagnetic waves was used to explain the reason behind the performance improvement of the rough SPR-based sensor. For TM-polarized light, the polarization can excite the SPR effects, or the electric field will affect the metallic surface along the x and z axes, while there are no magnetic fields in the two axes. The total intensity of the electric field along the *x*-axis (|E_x_|^2^) and *z*-axis (|E_z_|^2^) provides a good indicator for the summation of the LSPR strengths across the surface.

**Figure 15:**
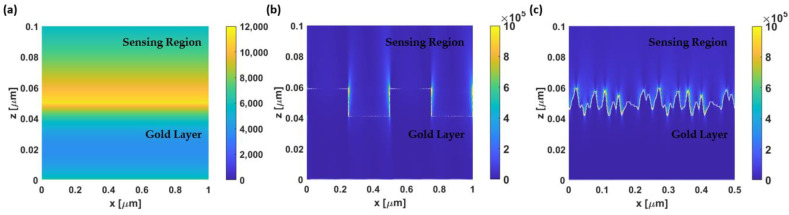
The plots of the total electric field intensity in the SI unit in the x-direction and z-direction (|E_x_|^2^ + |E_z_|^2^) on the gold-based SPR sensor with (**a**) an ideal smooth surface; (**b**) gratings structure with a gratings’ height of 24 nm, a gratings’ period of 500 nm, and a fill factor of 0.5; and (**c**) a rough sensing surface with *h* and *cl* of 9 nm and 8 nm, respectively (operating point ‘d’).

Figure 15 illustrates the electric field in the x-direction and z-direction for the TM-polarization case. Figure 15a–c illustrate the total electric field intensity of the SI unit, |E_x_|^2^ + |E_z_|^2^, in three different SPR-based sensor surfaces, including a theoretical smooth gold film, a gold grating structure, and a rough gold surface, respectively. For the uniform SPR-based sensor, the electric field intensity was evenly distributed along the gold surface with a maximum magnitude of 11.28 × 10^3^, as shown in Figure 15a. On the other hand, the total intensity of the electric field for a gratings sensor can reach up to 16.08 × 10^5^, which is a hundred times greater than the field intensity of the ideal smooth sensor case. Note that the color scales in Figure 15b,c have been adjusted to clearly show the electric field intensity across the entire surface (their maximum values could exceed the provided color bar limit). The gratings’ height, period, and fill factor of 24 nm, 500 nm, and 0.5 were selected according to the analyzed rough surface model.

Moreover, the electric field with the significantly high intensity only appears on the sharp edges of the gratings, while the intensity in the groove area ranges from 6.00 × 10^3^ to 30.00 × 10^3^. The rough surface in the operating point ‘d’ (*h* and *cl* of 12 nm) was chosen to investigate the magnetic field behavior due to the highest achieved *FoM*. The maximum electric field intensity obtained can be varied from approximately 14.92 × 10^5^ to 17.51 × 10^5^, depending on the randomly simulated surface. Even though the gratings and the rough surfaces produced similar electric field intensities, the roughness model provided significantly higher sharp edges, resulting in more LSPR hotspots and increased sensitivity.

## 5. Conclusions

The SPR-based sensor was responsive to changes in the plasmonic sensor surface. Therefore, a slight change in the surface structure, including the roughness, can strongly affect the sensor’s performance. This manuscript analyzes the effect of roughness on binding kinetic measurement for SPR detection by employing the RCWA and the Monte Carlo simulation on the constructed rough surface based on the Gaussian random surfaces method. The simulation structure consisted of two models: the nonsidewall BSA model, indicating the same amount of protein as the roughness changed, and the sidewall BSA model, where the protein quantity increased with the roughness. The convergence test on the number of required diffraction orders and simulated columns to represent the roughness and the SPR phenomenon was also applied to obtain the optimum environment for the theoretical study. The calculated reflectance spectra were then observed. Then, the performance factors were observed and calculated, including *S*, *n_0_sinθ_sp_*, *FWHM*, Δ*I*, *I_sp_*, and *FoMs*.

The surface roughness can improve, degrade, or deteriorate the SPR sensing performance due to the generated LSPR and the additional protein amount. The sensor can have an exceptionally high *S* with highly rough surfaces due to the distorted plasmonic dip structure, which reduced the *n_0_sinθ_sp_* and field enhancement effect from the surface roughness. However, utilizing the rough surface shows a tradeoff between the improved *S* and the crucial deterioration in the intensity detection capability. One suggested approach for the SPR application is to utilize the negative-sensitivity SPR detection at slight roughness levels. The reflectance spectra obtained from this region can increase the *S* to threefold of the ideal uniform gold sensor’s *S* while maintaining the *FWHM*, Δ*I*, and *I_sp_*. The operating points for the highest *FoM_1_* and *FoM_2_* enhanced the *FoMs* by 8.03 and 1.04 times compared to the sensor with a theoretically smooth surface. In addition, the roughness level could be utilized in other SPR measurement schemes, including intensity and phase detection. Further analysis indicates that the sensing improvement and degradation were primarily affected by the LSPR effect of over 70% in all operating cases. In conclusion, the knowledge of surface roughness can be employed to enhance the protein binding sensing performance of the SPR-based sensor.

## Figures and Tables

**Table 1:** The root mean square (RMS) roughness of metal film after different surface treatments.

Surface Treatment Techniques	RMS Roughness
Sputter Coating (No Treatment) [31]	1.4–2.5 nm
Thermal Annealing [32]	<1 nm
Chemically Grown Single-Crystalline Gold [35]	<1 nm
Chemical Polishing [37,38]	0.38 ± 0.05 nm
Helium Ion Beam [33]	0.267 nm
Mica Substrate Utilizing [36]	0.2 nm
Laser Ablation [34]	0.17 nm

**Table 2:** Shows the roughness parameters and plasmonic angles from the experimental results reported by Yang et al. [56] and the plasmonic angles from Figure 7.

Thermal Annealing Temoerature in °C	RMS of Roughness (nm)	Roughness Height (nm)	Experimental θ_sp_ (Degrees)	Simulated θ_sp_ Using the Proposed Method (Degrees)
100 °C	1.260	6.1	44.04	44.04
200 °C	0.906	3.7	43.94	43.93
300 °C	0.700	2.9	43.75	43.70
400 °C	0.415	1.5	43.65	43.56

**Table 3:** The quantitative performance parameters comparison between the surface plasmon resonance detection employed with a uniform and rough gold layer.

Quantitative Performance Parameters	Ideal Uniform Surface	*h* = 3 nm, *cl* = 10 nm (Operating Point ‘a’)		*h* = 10 nm, *cl* = 30 nm (Operating Point ‘b’)		*h* = 12 nm, *cl* = 20 nm (Operating Point ‘c’)		*h* = 9 nm, *cl* = 8 nm (Operating Point ‘d’)		*h* = 16 nm, *cl* = 15 nm (Operating Point ‘e’)	
		No Sidewall BSA	Sidewall BSA	No Sidewall BSA	Sidewall BSA	No Sidewall BSA	Sidewall BSA	No Sidewall BSA	Sidewall BSA	No Sidewall BSA	Sidewall BSA
*S* (rad/µm^2^)	122.59	−379.88	−379.88	85.31	85.31	996.11	1280.00	1191.13	1608.02	-	-
*n_0_sinθ_sp_* (unitless)	1.440	1.481	1.481	1.506	1.506	1.495	1.496	1.486	1.490	1.466 *	1.473 *
*FWHM* (rad∙RIU /µm)	0.41	0.58	0.58	0.79	0.79	0.66	0.65	0.67	0.67	0.77	0.75
Δ*I* (unitless)	0.90	0.88	0.88	0.47	0.47	0.13	0.15	0.10	0.14	0.001	0.003
*I_sp_* (unitless)	0.007	0.018	0.018	0.41	0.41	0.72	0.70	0.69	0.60	0.75	0.69
*FoM_1_* (unitless)	299.00	−654.97	−654.97	107.98	107.98	1509.26	1969.23	1777.80	2400.02	-	-
*FoM_2_* (unitless)	980.66	−1677.42	−1677.42	92.52	92.52	590.75	833.81	616.84	1020.33	-	-

* The *n_0_sinθ_sp_* of the operating point ‘e’ can still be determined. However, the reflectance spectrum of the model without a protein coating lost its plasmonic dip structure, resulting in an unidentified *S* and *FoM*.

**Table 4:** Shows the enhancement ratio of *S* and *FoMs* compared to an ideal uniform gold case and their effect in percentage due to the additional protein and wave effect from the surface roughness.

	*h* = 3 nm, *cl* = 10 nm (Operating Point ‘a’)	*h* = 10 nm, *cl* = 30 nm (Operating Point ‘b’)	*h* = 12 nm, *cl* = 20 nm (Operating Point ‘c’)	*h* = 9 nm, *cl* = 8 nm (Operating Point ‘d’)
*ER_S,roughness_*	−3.10	0.70	10.44	13.12
*ER_S,LSPR_*	−3.10 (100%)	0.70 (100%)	8.13 (77.87%)	9.72 (74.07%)
*ER_S,protein_*	0 (0%)	0 (0%)	2.31 (22.13%)	3.40 (25.93%)
*ER_FoM1,roughness_*	−2.19	0.36	6.59	8.03
*ER_FoM1,LSPR_*	−2.19 (100%)	0.36 (100%)	5.05 (76.64%)	5.95 (74.07%)
*ER_FoM1,protein_*	0 (0%)	0 (0%)	1.54 (23.36%)	2.08 (25.93%)
*ER_FoM2,roughness_*	−1.71	0.09	0.85	1.04
*ER_FoM2,LSPR_*	−1.71 (100%)	0.09 (100%)	0.60 (70.85%)	0.63 (60.45%)
*ER_FoM2,protein_*	0 (0%)	0 (0%)	0.25 (29.15%)	0.41 (39.55%)

## Data Availability

Not applicable.
